# Progress about the fibro-adipose vascular anomaly: A review

**DOI:** 10.1097/MD.0000000000037225

**Published:** 2024-02-16

**Authors:** Yi-Ran Sun, Ming-Li Zou, Si-Ming Yuan

**Affiliations:** a Department of Plastic Surgery, Jinling Hospital, Medical School of Nanjing University, Nanjing, Jiangsu, China

**Keywords:** diagnosis, fibro-adipose vascular anomaly, pathogenesis, treatment

## Abstract

Fibro-adipose vascular anomaly (FAVA) is a rare and complex vascular malformation associated with persistent pain, limb contracture, and even restriction of activity. However, the pathophysiology of FAVA remains unclear. Although FAVA is a benign vascular malformation, it is highly misdiagnosed and often thus undergoing repeated surgical resection and interventional sclerotherapy, resulting in worsening of symptoms and irreversible dysfunction. Therefore, aggressive diagnosis and treatment are essential. There are several different treatment options for FAVA, including surgical resection, sclerotherapy, cryoablation, drug therapy, and physical therapy. This article reviews the clinical manifestations, pathological features, pathogenesis, and treatment methods of FAVA.

## 1. Introduction

Fibro-adipose vascular anomaly (FAVA) is a rare congenital vascular malformation which is characterized by phlebectasia and fibrofatty tissue replacement of muscle, lymphatic involvement, and venous thrombosis.^[1]^ It was first reported in 2014 by Alomari et al at Boston Children Hospital and was included as a provisionally unclassified vascular anomaly in the 22nd International Society for the Study of Vascular Anomalies (ISSVA).^[2,3]^ The muscles of the extremities typically manifest as FAVA. It is often considered a slow-growing mass and its first symptom is mostly persistent and progressive pain. Owing to the above symptoms together with the lack of flow on ultrasound and anatomical location, FAVA has a high misdiagnosis rate. It often overlaps with other vascular malformations and tumors such as intermuscular hemangioma, angiolipoma, VM with muscle contracture and so on. Repeated surgical resection and interventional sclerotherapy lead to worsening symptoms and irreversible dysfunction, causing constant pain to the patient. Therefore, clear diagnosis and early surgery are extremely important for this disease. Our article provides a series of summaries of the clinical symptoms, pathogenesis, and treatment options of FAVA, hoping to provide reference guidelines for clinical treatment.

## 2. Diagnosis

The diagnosis of FAVA is based on the clinical and imaging findings. Pathological confirmation of the diagnosis is the gold standard for the disease.

### 2.1. Clinical manifestations

FAVA usually presents with limb pain and dysfunction which is caused by muscular pain involving the muscles affected by FAVA. Calves and forearms are the predominant sites of this complicated mesenchymal malformation.^[4]^ Deep intramuscular vascular malformation, along with diffuse fibrotic infiltration of the muscle and extension along the fascial planes, is the essence of FAVA. FAVA lumps are typically stiff, solid, and noncompressible on ultrasound. It is mostly located in the deep muscular layer with clear margins. The diagnosis generally revealed no skin abnormalities. Skin presentations such as skin lymphatic vesicles,^[5]^ capillary abnormalities, superficial vein dilatation, and hypopigmentation are only seen in a small percentage of individuals. In Addition, some individuals have paresthesia and localized swelling symptoms.^[6]^ Pain associated with FAVA is typically extremely persistent and has many different potential causes. The presence of widespread calf soreness points to fibrolipomatosis, a condition in which fibro-adipose tissue invades the muscle and causes abnormalities in muscule contraction or relaxation. Venous thrombosis, localized subcutaneous fibrosis, and other vascular lesions can result in focal discomfort.^[4]^ Neurological pain is another form of pain that mostly arises from the nerve and the scarring surrounding the nerve which is affected by FAVA.^[7]^ Joint contractures and motor dysfunction are prominent clinical signs of FAVA. Regardless of the degree of involvement, all patients with FAVA within the gastrocnemius have restricted foot dorsiflexion or equinus deformity, which further contributes to motor abnormalities. Motor dysfunction in patients may be affected by the location and degree of lesion invasion. Nevertheless, one of the potential reasons for motor abnormalities may be extremely persistent and severe discomfort in individuals. In addition, severe discomfort may cause the corresponding muscle to atrophy, which may result in motor abnormalities.

### 2.2. Imaging findings

#### 2.2.1. Doppler ultrasonography (US).

As a soft tissue disease, ultrasound and magnetic resonance imaging (MRI) are the most commonly used imaging tests. The preferred test for FAVA is ultrasonography for its noninvasiveness. However, ultrasonography cannot reliably detect deep lesions, thus MRI become a more commonly used FAVA test. Ultrasound, including grayscale and color Doppler, can be used to visualize lesion margins, echogenicity, the vascular system within the lesion, presence of blood clots, and formation of phleboliths and dilated blood flow patterns within the veins. Normal ultrasound of FAVA typically shows an uneven, hyperechoic solid intramuscular mass with destroyed venous passages.^[8]^ Large veins can be seen in some masses that extend into the subcutaneous tissue and occasionally have thrombosis because the FAVA is made up of fibro-adipose tissue and low-flow vascular abnormalities.^[9]^ Vein thrombosis and dilatation can be detected using the color Doppler in the subcutaneous tissue layer.^[10]^

#### 2.2.2. Magnetic resonance imaging (MRI).

The region that the lesion affects, the distribution, the signal signature, and the uniformity of the contrast enhancement pattern can all be determined by using MRI. On T1-weighted images, FAVA exhibits diverse signal intensities. On T2-weighted images, it exhibits moderately high-intensity, heterogeneous signal intensities.^[8]^ The muscle lesions are mildly increased following intravenous contrast injection. The distinguishing MRI findings of FAVA are regarded to be heterogeneous hyperintense T2, visible fat, dilated veins entwined within lesions, dilated subcutaneous veins, or fat overgrowth. It is also possible to claim that the muscles have been replaced by asymmetrical soft tissues, varicose veins, and dilated blood vessels. Fascial tail indications, or soft tissue masses that extend in a linear or conical form along the fascial border, may be a significant imaging hallmark of FAVA illness, according to research by Hu. published in 2022.^[11]^ Fascial tail indication helps differentiate FAVA from other venous abnormalities.

#### 2.2.3. X-ray.

X-rays are typically used to demonstrate bone changes. In most cases, soft tissue masses are visible on X-ray. Venous stones were visible in some instances. The structure of joints is typically normal.^[10]^ A crucial test to distinguish FAVA from VMs is needle phlebography, which is typically carried out as part of sclerotherapy.^[6]^ Aberrant veins in the lesion can be found via phlebography. Multiple aberrant venous branches in the lesion can be observed when contrast with FAVA is injected into the skin. The aberrant vein walls had the typical beaded appearance. Lesions are densely attached deep into the dermis, but the superficial layers of subcutaneous fat are usually not visible. The fat inside the lesion is usually tan. Outside the muscle, abnormal fibro-adipose tissue is seen along the fascial plane and neurovascular bundles. Vascular channels are significantly more numerous inside and surrounding the fat.^[10]^

## 3. Pathological features and pathogenesis

Histopathologically, FAVA is composed of fibro-adipose tissue as well as slow-flow vascular malformations (lymphoid and VMs) inside the muscles and surrounding subcutaneous tissues.^[12]^ A common venous component of FAVA is venous dilatation, which is often observed in the afflicted muscle or nearby subcutaneous tissue. Only a few patients develop modest subcutaneous lymphangiomas and subcutaneous venous abnormalities.^[5]^ In 2014, Fernandez-Pineda et al discovered that adipose tissue, conspicuous dense fibrous tissue, and clusters of thin-walled back-to-back veins with aberrant lymphovascular components make up the majority of FAVA mutations. In addition, there are a few additional supportive histological findings, such as the occurrence of variably muscularized veins, lymphoid or lymphoplasmacytic aggregates, and sporadic foci of osseous metaplasia.^[13]^ These include lymphoplasmacyte aggregation, skeletal muscle atrophy, and nerve entrapment.^[4]^

The 2 components of the immunohistochemical analysis of FAVA are aberrant lymphatic vessels and veins. Studies have indicated that platelet endothelial cell adhesion molecule-1 and CD34 are positive in aberrant veins, whereas D2-40 and prospero homeobox protein 1 are negative. CD34 was negative whereas platelet endothelial cell adhesion molecule-1, D2-40, and prospero homeobox protein 1 were positive in the aberrant lymphatic vessels. In all instances, aberrant vascular and fibrous tissues exhibit P-AKT, phosphorylated protein kinase, strain AK, Thymoma, and mTOR effectors p-S6K1 and *P*-4EBP1.^[14]^

**Figure 1.**
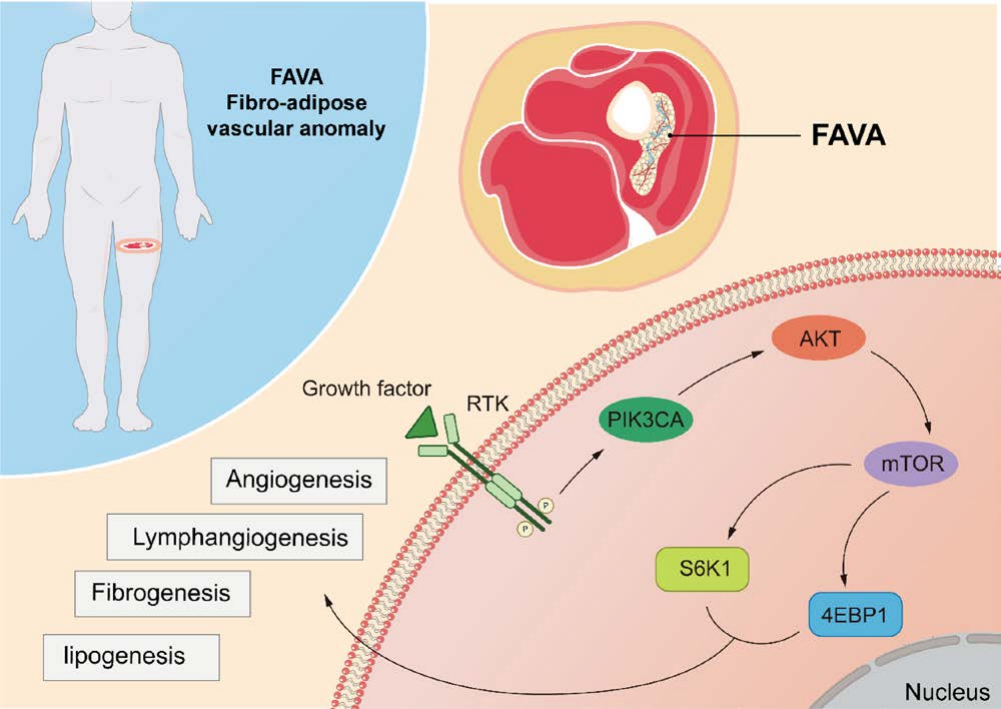
Pathological features of FAVA.

Luks et al^[15]^ first hypothesized that somatic mutations in PIK3CA are the cause of FAVA in 2015. Hori et al^[14]^ hypothesized in 2020 that FAVA is a PIK3CA-related overgrowth spectrum since somatic and mosaic cells gain functional mutations in the phosphatidylinositol-4,5-bisphosphate 3-kinase catalytic subunit alpha (PIK3CA) gene were discovered in FAVA. PIK3CA encodes the 110 kD catalytic subunit of phosphoinositide 3-Kinase (PI3K), a crucial lipid kinase that regulates metabolic signaling pathways and cell motility, proliferation, and survival. The PIK3CA pathway activates PI3K through different receptors, leading to the phosphorylation of AKT, thereby stimulating the activation of the mammalian target of rapamycin (mTOR). In addition, phosphorylated ribosomal protein S6 kinase 1 (S6K1) and eukaryotic translation initiation factor 4E-binding protein 1 (4EBP1) have stimulatory functions in vascular and lymphangiogenesis increasing protein synthesis and cell growth. Additionally, S6K1 and 4EBP1 are downstream targets of the phosphorylated form of mTOR, which promotes the growth of lymphatic and vascular vessels. In 2020, Restrepo et al^[16]^ detected PIK3C mutations in the passage fibroblasts and lymphoid endothelial cells of FAVA. Furthermore, PIK3CA mutations in fibroblast lines overly stimulate PI3K/AKT/mTOR signaling and downstream phosphorylation. This demonstrates that the PIK3CA pathway may also induce fibrogenesis and adipogenesis in FAVA in addition to angiogenesis and lymphangiogenesis. This finding was verified by a pathology report published in 2022.^[17]^ Hori et al^[18]^ discovered that the PIK3CA gene was not always linked to FAVA incidence in the same year. Some FAVA patients only find receptor tyrosine kinase hotspot mutations rather than PIK3CA mutations. FAVA mutations missing PIK3CA may result in changes in other 4EBP1 and S6K1-activating genes (Fig. 1).

## 4. Differential diagnosis

The most important methods for differential diagnosis are clinical manifestations and histology. The main differential diagnoses include venous malformation (VM), PTEN hamartoma tumor syndrome, Klippel–Trenaunay syndrome (KTS), and arteriovenous malformation (AVM).

### 4.1. Venous malformation

VM is a common vascular malformation composed of localized or diffuse abnormal veins.^[19]^ It often manifests as a local lump and can occur in any part of the body with a soft touch. The main pathological finding in VMs is the vessel wall. These lesions are usually seen as cavernous lesions, consisting of unorganized dilated channels and abnormal thin-walled structures, without any lymphatic components or infiltration of fibrous adipose tissue.^[13]^ The triangular fascial tail sign on MRI is helpful in distinguishing FAVA from VM.

### 4.2. PTEN hamartoma tumor syndrome

PTEN hamartoma tumor syndrome defines a spectrum of multi-system disorders caused by PTEN gene alterations, including Cowden syndrome, Bannayan Riley Ruvalcaba syndrome, PTEN-related proteus syndrome and Proteus-like syndrome and so on.^[20]^ The characteristic change in PTEN hamartoma tumor syndrome is the appearance of multiple hamartomas, commonly seen in the skin and digestive tract, which can manifest as papular changes in the facial skin, papillary changes in the oral mucosa, acrokeratosis, and gastrointestinal polyps. The most common clinical manifestation is mucosal involvement, which mostly affects the buccal and gingival mucosa and gives a characteristic “cobbled” appearance. PTEN hamartoma tumor syndrome is an unsealed nodular mass that consist of a mixture of adipocytes and fibrous tissue, as well as thick walled arteries, venous channels and small vessels.^[13]^ Tissue biopsy is the gold standard for distinguishing between low-flow PTEN hamartomas and FAVA lesions.

### 4.3. Klippel–Trenaunay syndrome

KTS is a complex disorder that includes capillary, lymphatic, and VMs with limb.^[21]^ The clinical manifestations of KTS include skin capillary malformations, abnormal growth of superficial and deep veins, and limb asymmetry. Venous abnormality is the main cause of vascular malformations in this disorder. Unlike FAVA, KT syndrome does not involve abnormal fat hyperplasia.

### 4.4. Arteriovenous malformation

Arteriovenous malformation is an abnormal vascular lesion composed of blood vessels of various sizes, with 1 or more direct connections between arterial and venous circulation.^[22]^ The clinical manifestations are mainly characteristic pulsation, tremor, temperature increase in the lesion, local pain, ulceration or repeated bleeding. Severe cases due to long-term hemodynamic abnormalities can lead to heart failure. The imaging methods for detecting arteriovenous malformations include MRI and magnetic resonance angiography. MRI displays signal gaps consistent with rapid flow, whereas FAVA cannot. The lumen was irregularly dilated. The thickness of the tube wall in arteriovenous malformation varies, but most of them are thick while the vascular wall is vitreous and mucoid. In arteriovenous malformations, there is abnormal direct connectivity between the small arteries and veins, including a mixture of malformed blood vessels.^[13]^

## 5. Treatment

Treatment options for FAVA include medication, physical therapy, surgery, cryoablation therapy, sclerotherapy, combination therapy, and more.

In 2014, surgical excision of the lesions was discovered to be successful.^[4]^ Surgery is considered a reliable and effective option and may be the only way to control FAVA in the long-term. However, surgery may increase the risk of bleeding or aggravating symptoms when treating abnormalities. Surgery is also linked to partial resection and recurrence at the same time. Resection was deemed the best course of action for treating symptomatic lesions and developing muscular contractures in 2020 by Hori et al.^[14]^ Surgical resection usually includes subtotal resection, neurolysis, capsulotomy, and tendon lengthening (optional). Removal of large, extensive lesions requires muscle resection, so tendon displacement or tendon extension can be used to restore function, or free-band vascular muscle displacement can be used to restore muscle function. In the literature, amputation is frequently used as a treatment for limbs that have severe vascular abnormalities.^[23]^ Amputation may be a realistic salvage option if the lesion is deep or if there remains unbearable, intractable pain after surgical removal.

Sclerotherapy injections are typically utilized to treat individuals with FAVA who have received an incorrect diagnosis because the procedure is a standard treatment for VMs. In 2014, percutaneous sclerosing therapy was temporarily helpful in temporarily reducing pain, but the horseshoe contracture did not resolve and needed surgical resection and Achilles tendon lengthening.^[4]^ In 2016, sclerotherapy has become the mainstay of treatment for the low-flow malformed portion of FAVA.^[24]^ However, sclerotherapy is typically ineffectual for the remaining FAVA. It only is used to deal with small, localized lesions and subcutaneous regions. On the other hand, failure to respond to sclerotherapy may point to FAVA diagnosis.^[25]^

Cryoablation therapy was first proposed by Shaikh et al in 2016.^[26]^ Cryoablation treatment has been found in studies to be useful for reducing patient pain. Inflammatory fibro-adipose tissue may have a direct cytotoxic impact or axonal degeneration may be the cause of cryoablation capacity to reduce FAVA discomfort. Percutaneous cryoablation has few side effects and a quick recovery time as advantages. But at the same time, the degree of ablation cannot be properly regulated, which might result in muscle injury around the site of ablation and discomfort there. According to Ramaswamy research in 2019^[20]^, cryoablation has replaced sclerotherapy as the standard treatment for FAVA and is both safe and efficient for treating low-flow vascular malformations.^[27]^ It was established in 2020 that this technique has a larger ablation area and causes less trauma than traditional surgery.^[28]^ In 2021, Lipede et al proposed that cryoablation may be less effective in patients with extensive disease or established contractures.^[25]^ However, owing to the small number of patients receiving cryoablation, the conclusions of the current study do not guarantee its safety.

Drug therapy has emerged as a novel strategy for treating FAVA owing to developments in molecular biology. In 2017, Erickson et al reported drug regimens for 2 patients with FAVA. Both patients experienced severe pain and impairment, and sirolimus treatment significantly improved their conditions.^[29]^ Sirolimus SRL, also known as rapamycin, is a new macrolide immunosuppressed extract from Streptomyces hygroscopicus that is, similar to tacrolimus. Sirolimus, an mTOR inhibitor, prevents the phosphorylation of aberrant blood vessels in fibro-adipose tissue and FAVA, which prevents the formation of blood vessels and lymphatic vessels at lesions. However, there is a chance that the lesion will return after quitting treatment, therefore low-dose maintenance therapy must be taken into account later on.

There have not been many reports on physical therapy or compression therapy, but Cheung study found that patients who received treatment showed no improvement.^[10]^ In 2020, Stillo et al^[28]^ proposed through a case study that absolute ethanol embolization plus surgery is a safe, effective, and long-term treatment for FAVA skin lesions. Additionally, surgery combined with medication therapy can accomplish the dual goals of simultaneously curing the lesion dramatically and momentarily reducing pain (Table 1).

**Table 1:** Treatment of FAVA.

Treatment	Advantage	Disadvantage
Surgery	Treat symptomatic lesions and developing muscular contractures	Raise the risk of bleeding or aggravating symptoms
Sclerotherapy	Reduce pain temporarily	Unresolve the horseshoe contracture
Cryoablation therapy	Recover quickly	Might result in muscle injury around the site of ablation
Durg therapy	Without surgical or anesthesia risks	Lesion return after quitting treatment

## 6. Conclusion

In conclusion, the FAVA approach improves the new classification of vascular malformations and lowers the likelihood of a false positive. Imaging studies and clinical signs are typically used to confirm the diagnosis of FAVA. Ultrasound is frequently used as the preferred adjunct to FAVA and is frequently used for preoperative evaluation or intraoperative localization of FAVA. MRI is primarily used to observe the specific size of the lesion, clarify the relationship between the lesion and the surrounding soft tissue, and is frequently used to distinguish it from other venous diseases. There hasn’t been much research done on FAVA pathophysiology, specifically how the PIK3CA pathway is activated. Other statements have been made in recent years, but more investigation is required. There are numerous treatment options available today for FAVA, and each 1 has its own indications, benefits, and drawbacks. To select the most effective course of action, it is essential to carefully weigh all relevant elements. The future standard of care for FAVA treatment may be combination therapy. In conclusion, FAVA requires more investigation of its etiology as a recently suggested and often misunderstood disease in order to provide patients with more precise therapy.

## Acknowledgments

The authors thank Dr Hai-Yan Zong and Yong Chen for their help in polishing the article.

## Author contributions

Writing—review & editing: Yiran Sun, Mingli Zou, Siming Yuan
